# Evaluation of the Antimicrobial Resistance Surveillance System in Sentinel Sites in Cameroon

**DOI:** 10.7759/cureus.40779

**Published:** 2023-06-22

**Authors:** Daniele Sandra Yopa, Priscillia Anya, Patricia Mendjime, Tatiana Elouga, Emmanuel Nnanga-Nga, Georges Nguefack-Tsague

**Affiliations:** 1 Department of Public Health, University of Yaounde 1, Yaounde, CMR; 2 Epidemiology, Cameroon Field Epidemiology Training Program, Yaounde, CMR; 3 Epidemiology, Higher Institute of Science and Health Technologies, Yaounde, CMR; 4 Department of Pharmacology, University of Yaounde 1, Yaounde, CMR

**Keywords:** sentinel sites, cameroon, evaluation, attributes, surveillance, antimicrobial resistance

## Abstract

Background

The purpose of antimicrobial resistance (AMR) surveillance is to guide clinical decision-making, characterize trends in resistance infections, and provide epidemiological data to study the impact of AMR on health and the effectiveness of control measures in health facilities and the community. To do this, regular and relevant assessments of standardized AMR surveillance systems are essential to prioritize threats and improve their performance and cost-effectiveness. The scarcity of data and the absence of a local and national strategy on the surveillance of antibiotic resistance in Sub-Saharan Africa and even more so in Cameroon do not allow an effective response to be carried out against the scourge. This gap led us to conduct a study on the evaluation of the attributes of the antibiotic resistance surveillance system in Cameroon.

Methodology

We conducted a descriptive, cross-sectional study over a period of one year from January to December 2021. The study was conducted in the sentinel sites of surveillance in Cameroon, namely, those of the Centre, South-West, Littoral, and North regions. Using structured questionnaires and a pre-established and pre-tested interview guide, we collected data that allowed us to assess a surveillance system’s quantitative and qualitative attributes according to the CDC guidelines. Scores were assigned based on the different questionnaires to assess the attributes of the AMR surveillance system.

Results

Of the evaluated attributes, it appears that although the system is useful (88.9%, i.e., a score of 2), and has good completeness of data transmission (98.9%, i.e., a score of 3), it is not simple (64.3%, i.e., a score of 1), not stable (58.6%, i.e., a score of 1), not acceptable (58.6%, i.e., a score of 1), and presents poor data quality (11.05%, a score of 1).

Conclusions

The AMR surveillance system in Cameroon is useful with good completeness. However, many other attributes have poor performance, indicating the importance of improving the antimicrobial surveillance system.

## Introduction

Antimicrobial resistance (AMR) was declared by the WHO as one of the 10 global public health threats facing humanity [1]. Some studies have shown high levels of resistance to several bacterial infections in many countries around the world [2-5]. This fundamental threat raises fears that some infectious diseases will become incurable in a post-antibiotic era [6]. As a starting point for the response to AMR, the WHO and global health action today encourage strategic, coordinated, and sustained efforts in each country to develop a national action plan for good, effective AMR surveillance and antimicrobial stewardship [7-10]. The Global Antimicrobial Resistance Surveillance System (GLASS) launched by the latter in 2014 represents a standardized approach to surveillance based solely on epidemiological, clinical, and population-based data [2,3,6,7]. GLASS collects AMR-based data primarily in the form of surveillance based on patient samples [3,11], including blood, urine, stool, urethra, and cervical specimens from designated laboratories for clinical purposes [6].

The purpose of AMR surveillance is to guide clinical decision-making, characterize trends in resistance infections, and provide epidemiological data to study the impact of AMR on health and the effectiveness of control measures in health facilities and the community [6,12,13]. To do this, regular and relevant assessments of standardized AMR surveillance systems are essential to prioritize threats and improve their performance and cost-effectiveness. It is also important to ensure that they are fit for purpose and that all actors understand and take responsibility. Valid surveillance can be incorporated into cross-national longitudinal studies to track changes in resistance over time in different domains [6,7,10]. The ability of surveillance systems to accurately describe the characteristics of AMR is important for this public health issue [9,12].

The surveillance strategies before their design validation must take into account the priorities of the country, economic constraints, demographics change, resources, clinical practices, and treatment of diseases.

The European Centre for Disease Prevention and Control was the first to report AMR surveillance in 2014 [7]. With this in mind, several types of surveillance assessment tools exist depending on the target to facilitate the implementation of these tools [8,12]. The multiple attributes of tools allow looking at technical aspects, while others assess processes and systems. With the “One Health” approach, which is defined as the collaborative effort of multiple disciplines to achieve optimal health for people, animals, and the environment, a good assessment of integrated AMR surveillance helps to better situate decision-makers and actors on what needs to be done to improve behavior toward antibiotics in the human, animal, and environmental health sectors [7,10,14].

Due to economic constraints, the choice of antibiotics in most parts of Africa including Cameroon is generally not based on knowledge of bacterial susceptibility. A literature review of the implementation of AMR surveillance in the WHO African region found that two countries, Ethiopia and South Africa (4.3%), have implemented national AMR plans while seven (14.9%) other countries only have infection prevention and control policies. Four of these countries are from East Africa (Tanzania, Zimbabwe, Ethiopia, and Kenya), two are from Southern Africa (Lesotho and South Africa), and one is from West Africa (Ghana). Furthermore, no African country has a national surveillance system that regularly generates representative and solid data on antimicrobial use and resistance [8].

Several organizations have developed their own approaches to conducting evaluations of AMR surveillance systems and drawing relevant recommendations. This results in influencing decision-making regarding antibiotic management. Preserving the effectiveness of antibiotics while guaranteeing universal access is today an ethical obligation [7].

To effectively combat AMR and based on the recommendations of the World Organisation for Animal Health and the Joint External Evaluation of the International Health Regulations (2005), in 2018, Cameroon adopted its national action plan to combat AMR [15]. The document had six strategic objectives, and the second objective of the document was to strengthen the knowledge and evidence base through surveillance and research. With this in mind, one of the priority actions was to set up an AMR surveillance system. Although there is no evidence of the regional strategy for the surveillance of antibiotic resistance in Sub-Saharan Africa, there is sentinel surveillance of AMR that has been put in place in Cameroon in 2021. This study was performed to describe the structure and assess the attributes of the AMR surveillance system after one year of implementation.

## Materials and methods

Study design and study population

We conducted a cross-sectional, descriptive study. We conducted our assessment of the surveillance system over a period of one year from January to December 2021. Our study was conducted in the various sentinel surveillance sites in Cameroon, namely, those of the Centre, South-West, Littoral, and North regions.

**Figure 1:**
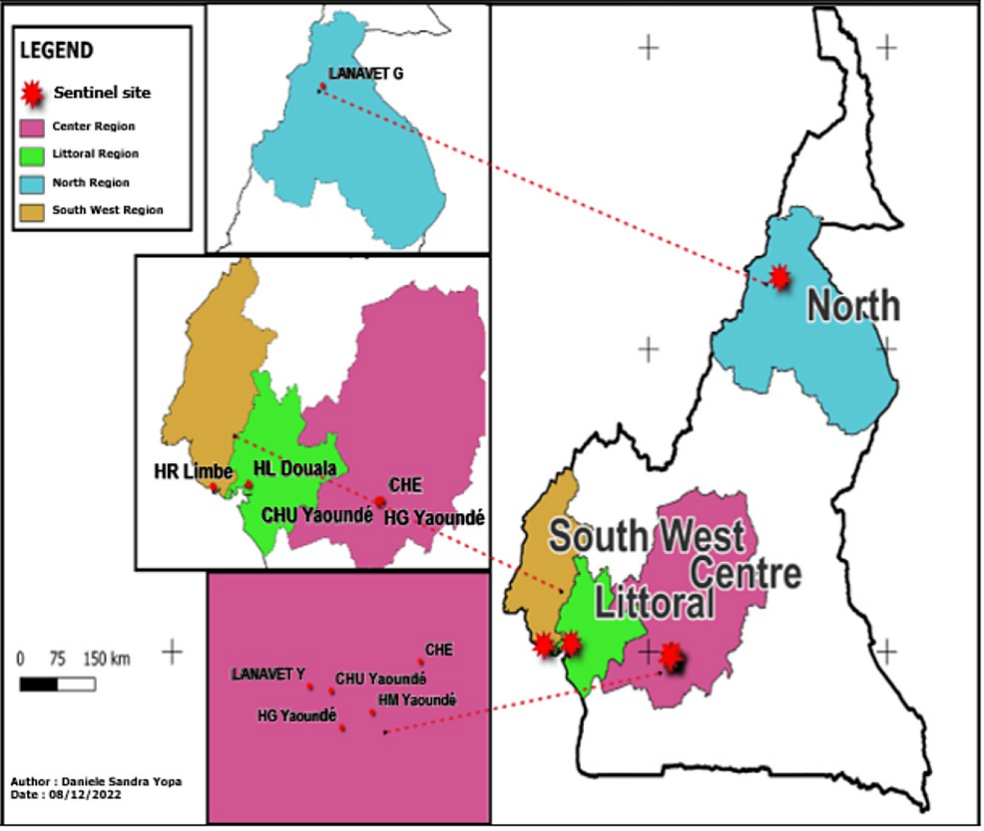
Mapping of sentinel sites in Cameroon.

Our population was made up of actors involved in AMR surveillance at sentinel sites. We included any actor involved in AMR surveillance in any of the AMR surveillance sentinel sites. Those actors are either the focal person of the different sentinel sites for AMR surveillance or their substitutes. Considering that aspect, the selection of the people interviewed was made by a reasoned choice of the structures and people involved in AMR surveillance. We also interviewed the focal persons of the sectors involved in the surveillance of the AMR at the strategic level in Cameroon. Figure 1 presents the mapping of sentinel sites in Cameroon.

Sources of information

We used data from the WHONET tool, surveillance data from the field survey of actors, and used documents such as notification forms, the national guide to AMR surveillance, National Action Plan against AMR. WHONET is an application for the management and analysis of microbiology laboratory data with a particular focus on AMR surveillance.

Selection of sites and interviewees

We performed an exhaustive sampling of sentinel sites for AMR surveillance, which included Yaoundé University Hospital Center (CHUY), Essos Hospital Center (CHE), Yaoundé General Hospital (HGY), Military Hospital of Yaoundé (HMY), Laquintinie Hospital of Douala (HLD), Limbe Regional Hospital (HRL), and National Veterinary Laboratories of Yaounde and Garoua (LANAVET). We interviewed the surveillance focal points of the various coordination structures at the strategic level and the actors in charge of surveillance in the various sentinel sites in Cameroon, namely, the focal point of the Ministry of Agriculture and Rural Development (MINADER), the one of Ministry of Public Health (MOH), and the one of the Ministry of Livestock, Fisheries and Animal Industries (MINEPIA) to have the structure of the surveillance system of the AMR in Cameroon.

Data collection tools

We developed a pre-established and pre-tested structured questionnaire to administer during the interviews of managers and personnel in charge of surveillance AMR. We also designed a semi-structured questionnaire that was administered only to the focal persons of the three sectors (MINEPIA, MINADER, and MOH) for the coordination of surveillance. This enabled us to assess our quantitative and qualitative attributes, namely, usefulness, acceptability, representativeness, and simplicity of the surveillance system, which are the attributes derived from the CDC guidelines [16]. Two data collection sheets were made to collect our data.

Procedure

After obtaining administrative authorizations from the hospitals hosting the AMR surveillance sentinel laboratories, we obtained an appointment with each of the actors involved in surveillance to administer our structured questionnaire. On the day of the meeting with each of the actors, we took care to explain the relevance of our study and obtained their consent. After obtaining informed and written consent, we collected the data. It took us less than 15 minutes. We then encoded the data. Attributes were assessed according to the CDC guidelines from Robert German’s 2001 task force [17]. For each of the questions evaluating the attributes of the surveillance system, we set a score to define the level of validity of the said attributes. The table presented in the Appendices summarizes the various questions addressed for the evaluation of the different attributes.

Usefulness

The evaluation of utility describes the impact of the information produced by the system on public health decisions. Usefulness was assessed by seven indicators. To judge the usefulness of the surveillance system, we calculated the frequency of the “Yes” answers obtained. If the frequency was less than 70%, we assigned a score of 1. If it was above 70%, we assigned a score of 2 (score 1 = not useful; score 2 = useful).

Simplicity

The simplicity assessment allowed us to determine if the structure of the surveillance system was efficient and easy to use. The system was considered simple if its structure involved a limited number of actors, if the definition of cases was clear and precise, and if the procedures for collecting and transmitting data were rapid and standard. Simplicity was evaluated by height indicators. To judge the simplicity of the surveillance system, we calculated the frequency of “Yes” answers obtained. If the frequency was less than 70%, we assigned a score of 1. If it was greater than 70%, we assigned a score of 2 (score 1 = not simple; score 2 = simple).

Stability

The stability assessment allowed us to determine if the surveillance system had experienced interruptions and if it had the material and human resources necessary for its operation. The system was deemed stable if it had not experienced any breaks in operation, and if it had qualified personnel and the appropriate equipment. Stability was assessed using 10 indicators. To judge the stability of the surveillance system, we calculated the frequency of the “Yes” answers obtained. If the frequency was less than 70%, we assigned a score of 1. If it was greater than 70%, we assigned a score of 2 (score 1 = not stable; score 2 = stable).

Acceptability

The evaluation of acceptability allowed us to know the will of the actors involved in surveillance to participate in the AMR surveillance process and to notify the health results obtained. Acceptability was assessed with five indicators. To judge the acceptability of the surveillance system, we calculated the frequency of “Yes” answers obtained. If the frequency was less than 70%, we assigned a score of 1. If it was greater than 70%, we assigned a score of 2 (score 1 = not acceptable; score 2 = acceptable).

Data Quality

Data quality reflects the completeness and validity of the data recorded in the AMR surveillance system. The values ​​of the recorded data were compared to the “true” values ​​through an examination of 10 datasets that we selected by balloting. We conducted a draw without replacement to identify the days, and another draw without replacement to identify the months. The draw pairs constituted the dates. For example, if the first day draw was “15” and the first month draw was “6,” our first dataset to examine would be “June 15, 2021.” Data quality was assessed using nine indicators. To judge the quality of the data from the surveillance system, we calculated the frequency of “Yes” answers obtained. If the frequency was less than 50%, we assigned a score of 1. If it was between 50% and 80%, we assigned a score of 2. If it was greater than 80%, we assigned a score of 3 (score 1 = poor quality; score 2 = ​​average quality; score 3 = good quality).

Completeness of Transmission of Reports

Assessing the completeness of data in the surveillance system consisted of ensuring that those involved in the surveillance reported the monthly surveillance data. To judge the completeness of the transmission of the reports of the surveillance system, we calculated the percentage of monthly reports sent to the NPHL during the year 2021. The number of reports expected for the whole year was 12 per site. If this frequency was less than 50%, we assigned a score of 1. If it was between 50% and 90%, we assigned a score of 2. If it was greater than 90%, we assigned a score of 3 (score 1 = low completeness; score 2 = average completeness; score 3 = good completeness).

Data entry and analysis

The data collected were compiled. Scores were assigned to categorize the surveillance system. All collection sheets were saved in a database designed using Excel 2016 software. The data were then analyzed using Excel 2016 spreadsheet software. Categorical data were presented in the form of frequencies and percentages. The results were presented in the form of graphs, tables, and figures. We used QGIS software 3.22.10 to map the different sentinel sites in the country.

Ethical considerations

The study obtained the approval of the Institutional Ethical Review Board of the Faculty of Medicine and Biomedical Sciences of the University of Yaoundé 1 (approval number: #121/UYI/FMSB/VDRC/DAASR/CSD). Authorizations were then obtained before the administration of questionnaires. Informed consent forms were signed by each participant. Confidentiality was maintained in the handling of service statistics and archived data.

## Results

Distribution of respondents according to the sentinel sites

**Figure 2:**
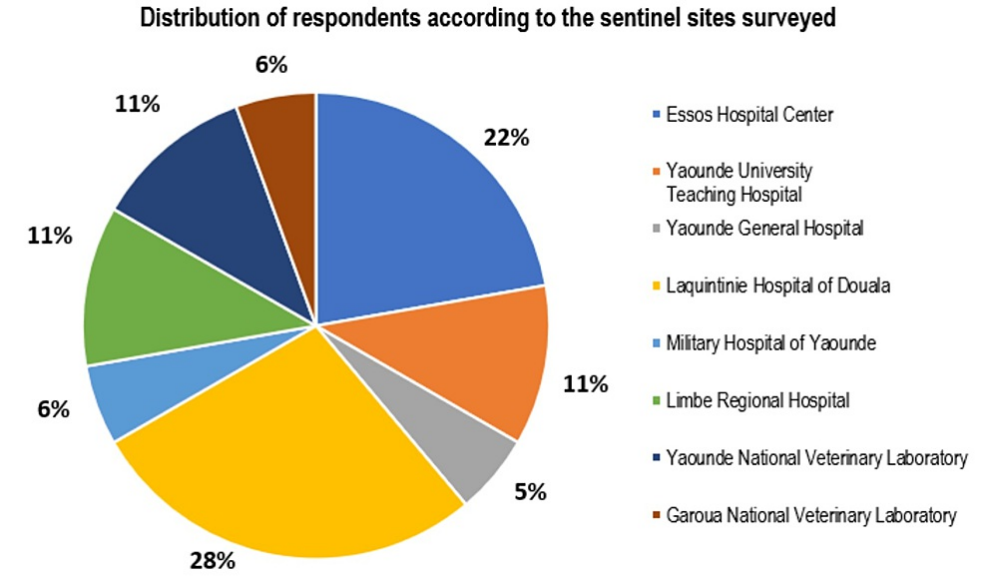
Distribution of respondents according to the sentinel sites surveyed.

The study included 18 AMR sentinel surveillance key actors in eight sentinel surveillance sites, namely, CHE, the CHUY, HGY, HLD, HMY, HRL, LANAVET Yaoundé, and LANAVET Garoua, as well as an actor of the Department of Veterinary Services. All of the said actors play a role in the detection and notification of AMR cases. Among the 18 sentinel site actors, nine (50%) are formally designated and trained AMR surveillance focal points. HLD recorded the greatest number of respondents (28%), and the HGY the fewest respondents (5%). Figure 2 presents the distribution of respondents.

Sociodemographic characteristics of the population

Our sample was made up predominantly of the female gender, in particular 11 (61%). Our sample was mostly made up of people between the ages of 31 and 40. The minimum and maximum ages were 26 and 51 years, respectively, and the median age 27.4. Our sample consisted of 15 (83%) and three (17%) people involved in the human and animal sectors, respectively. Of our respondents, eight (44.4%) were biologists, biological engineers, and medical-sanitary engineers; six (33.3%) were medico-sanitary technicians; two (11.1%) did not provide answers; and two (11.2%) were medical doctors and veterinarians, respectively. The attributes of the surveillance system assessed were usefulness, simplicity, stability, acceptability, data quality, and completeness.

System structure and operation

A total of eight AMR monitoring sentinel sites are all functional in four regions across the country since January 2021. Among the sites, there are six human health laboratories and two animal health laboratories. Apart from the designated focal point for monitoring, an additional person can most often detect and report AMR data. Human health surveillance of AMR is largely passive. Priority samples are collected at diagnostic laboratories for the detection of bacterial agents responsible for community or nosocomial infections. In animal health, surveillance is active and passive, with AMR data collection in intensive and semi-intensive farms. Environmental health is not yet involved at the operational level in the routine activities of AMR surveillance. The target bacteria under surveillance are defined by GLASS for global AMR surveillance. Any case meeting the AMR case definition in accordance with the national AMR monitoring guide is reported by site actors first in the laboratory registers. Then, the data is entered into the WHONET platform which is a standard tool and used for AMR monitoring in each of the sites. There is no interconnection between the sites’ databases. Each sentinel site focal point then sends the monthly surveillance databases by email to the Cameroon NPHL. Data should be transmitted by the second of each month. The NPHL the National Coordination Centre for AMR surveillance at the country level. It works directly with sentinel sites. It is in charge of consolidating all databases and analyzing them for a national presentation of surveillance data. It provides feedback to sentinel sites on the quality of the data analyzed.

System usefulness

The usefulness indicator of the system was 88.9%, i.e., a score of 2. The system is said to be “useful.” All of the people interviewed believe that the pilot system put in place makes it possible to monitor the AMR profile, identify the emergence of new resistant strains, and detect clusters of AMR cases early. For 14 (93.3%) participants, the surveillance system makes it possible to control the use and prescription of antibiotics in human health and two (66.6%) for the prescription of antibiotics in animal health. Overall, 94.4% (17/18) of the respondents think the data from AMR surveillance contributes to decision-making. For 15 (83.3%) participants, the system contributes to the strengthening of AMR detection capacities by laboratories.

System simplicity

The system’s simplicity indicator was 64.3%, i.e., a score of 1. The system is said to be “not simple.” Sixteen participants out of 18 (88.9%) find that the tasks to be performed as part of the surveillance are simple and easy to understand. A total of 10 (55.6%) respondents clearly stated the suspected AMR case definition, while 12 (66.7%) respondents clearly stated the confirmed AMR case definition. Concerning the handling of the WHONET software for data entry and analysis, nine (50%) of the respondents declared that the latter is easy to handle. Data entry into the software took less than 10 minutes for 10 (55.6%) of the participants. The next level of data transmission, which is the NPHL, was known to 15 (83.3%) of the participants. Ten (55.6%) participants knew the monthly deadline for data transmission which is the second of each month.

System stability

The system stability indicator was 58.6%, i.e., a score of 1. The system is said to be “not stable.” All 15 participants from the human health sector (83.3%) reported having staff in charge of AMR surveillance available, while participants from the animal sector reported not having permanent staff 24 hours a day. Among the eight focal points interviewed, five declared being assisted by other actors for AMR surveillance in the sentinel sites. Of the 18 participants, 14 (77.8%) were trained in AMR case detection and reporting. In total, 13 (72.2%) participants were trained in the use of WHONET. All sentinel sites have an electronic copy of the national action plan and the national AMR control guide. The notification forms validated in the national AMR control guide are not yet used in the sentinel sites. For reporting, sentinel sites use bench forms and registers dedicated to such surveillance. Regarding the availability of IT tools for data entry into WHONET, seven sentinel sites reported having a computer, cable, and software for AMR surveillance. Only the Douala General Hospital has a stable internet connection for the transmission of data to the NPHL. With regard to the specimens collected, no sentinel site has experienced a break in operation since the implementation of the AMR surveillance system.

System acceptability

The system acceptability indicator was 58.6%, i.e., a score of 1. The system is not said to be “acceptable.” Among the people surveyed, 13 (72.2%) think that the amount of information to be provided in the notification form is acceptable. Twelve (66.7%) believe that the information collected is used for the system. Thirteen (72.2%) said that no surveillance meeting was held between the different sites performing the surveillance and the coordination structure. Thirteen (72.2%) respondents said they found an interest in participating in AMR surveillance. Nine (50%) of 18 respondents said that AMR surveillance was not part of their daily activities.

Data quality assessment

The data quality indicator of the system to be evaluated was the completeness of data filling. It was 11.05%, i.e., a score of 1. The quality of the system’s data is said to be “poor.” The completeness of data transmission was calculated based on the number of monthly databases transmitted by each of the sentinel sites to the NPHL. We had access to data from six sentinel sites out of the eight expected (four human sites, and two animal sites).

The completeness of data transmission was 98.9%, i.e., a score of 3. The system has good data transmission completeness. As shown in Table 1, the authors tried to summarize the performance of each surveillance attribute.

**Table 1:** A summary of the performance of surveillance attributes and indicators in the sentinel sites of Cameroon.

Attributes	Score	Performances
Usefulness	2	System is useful
Simplicity	1	System is not simple
Stability	1	System is not stable
Acceptability	1	System is not acceptable
Data quality/Completeness of data filling	1	System has a poor completeness of data filling
Completeness of data transmission	3	System has a good completeness of data transmission

## Discussion

The objective of this part of our study was to assess the attributes of the AMR surveillance system between January 2021 and December 2021 in Cameroon. Of the eight attributes to be assessed, only timeliness was not assessed due to a lack of access to the NPHL. Of the seven other attributes evaluated, it appears that although the system is useful (88.9%, i.e., a score of 2) and has good completeness (98.9%, i.e., a score of 3), it is not simple (64.3%, i.e., a score of 1), not stable (58.6%, i.e., a score of 1), not acceptable (58.6%, i.e., a score of 1), and presents poor data quality (11.05%, a score of 1). According to the WHO Initial Implementation Manual for the Global Antimicrobial Resistance Surveillance System, the number of AMR surveillance sites is one of the indicators categorized as Public Health Priorities Targeted by Surveillance [9]. Thus, a country that has a weak surveillance system provides only partial data that does not reflect the true AMR situation in the country and cannot succeed to stop this scourge. Still, according to the aforementioned WHO document [9], the quality of the data and respect for the deadlines for the submission of the reports are essential. Not having been able to evaluate the timeliness in our study, it is important to wonder about the delays in the transmission of the data, which were classified as bad in this study.

In our study, the bad performance of AMR surveillance sites can be explained by the fact that the implementation of the national AMR surveillance plan is recent in Cameroon, as well as by the fact that there were no prerequisites of associations or structures that collected data on AMR. Indeed, the national action plan to fight against AMR was only published in May 2018 [15]. With regard to data quality, the number of periodic reports well-filled before transmission to the higher level in 2021 was less than 50%. Such data do not allow us to understand the complete state of AMR in Cameroon. The cause of this poor quality of data may be due to the lack of training of the people assimilated to the focal points. In fact, in our study, we interviewed 18 key actors, and among them, only nine (50%) were formally designated and trained for AMR surveillance. Lack of staff training is a handicap that can impact several attributes of the AMR surveillance system [10,15,18].

On the other hand, the type of surveillance used does not provide a real picture of the scale of the challenge to provide an appropriate response. The low number of laboratories providing surveillance and the lack of a technical platform and logistical means to ensure optimal detection of germs under surveillance could also explain the low quality of the data and the low stability of the system. This problem of limited capacity has also been highlighted by Shah et al. in South-East Asia [8]. With the example of France, we can see that long before the establishment of the national mission of surveillance of resistance to antibiotics and healthcare-associated infections in urban areas and medico-social establishments, there were structures that provided surveillance of antimicrobials, although this surveillance was not structured, and it served as the basis for the national mission. Thus, for 2018, the results presented in the study on AMR surveillance piloted by Santé Publique France were based on more than 400,000 antibiograms performed by 742 laboratories in 11 metropolitan regions [19]. Many networks benefiting from technical and logistical support are established for the surveillance of AMR but are very specific to certain germs and pathologies such as malaria, HIV, and tuberculosis [2].

Study limitations

The study was conducted just one year after the effective implementation of AMR surveillance in sentinel sites. We think that the delay is not sufficient to perform a good assessment of this surveillance system. Moreover, we have not selected all indicators to have a good overview of the performance of all the attributes of the AMR surveillance system.

## Conclusions

The purpose of this study was to evaluate the attributes of the AMR surveillance system. It emerges from the evaluation of eight sentinel sites, six for the human health sector and two for the animal health sector, which are actively involved in AMR surveillance. Although the environmental health sector is considered in the national plan and the national AMR control guide, it is not yet involved in field surveillance activities. The system is considered useful, and it has good completeness in data transmission. However, many other attributes have poor performance indicating the importance of improving the antimicrobial surveillance system. It is, therefore, important to strengthen the AMR surveillance system.

## Appendices

**Table 2:** Different questions asked for the assessment of the surveillance system attributes concerning antimicrobial resistance (AMR).

Differents indicators		
Usefulness indicators		
01	Does the system track resistance profiles?	
02	Does the system allow early detection of a cluster of AMR cases? Does the system allow early detection of a cluster of RAM cases? Is it helping to improve the control of antibiotic use?	
03	Is it helping to improve the control of antibiotic use?	
04	Does the data help inform decision-making?	
05	Does the data produced make it possible to identify the emergence of new resistant strains?	
06	Does the data producer make it possible to improve medical prescriptions in human health? (This question is intended for actors in the human health system)	
07	Do the data produced make it possible to improve the use of ATBs in livestock farming? (This question is intended for actors in the animal health system)	
Simplicity indicators		
01	Is the description of the tasks simple and understood by all the actors?	
02	Do staff clearly state the AMR suspect case definition according to the PAN-AMR? (Let the participant give the definition)	
03	Do staff clearly state the confirmed case definition of AMR according to the PAN-AMR? (Let the participant give the definition)	
04	Is the notification form or register simple to fill in?	
05	Is the WHONET software easy to handle according to the participant?	
06	Is the time needed to fill in the data in the WHONET less than or equal to 10 minutes?	
07	Do staff know the next level of data transmission? (Allow the participant to answer and mark “Yes” if they answer the following question correctly)	
08	Do you know the monthly delay for transmitting data to the next level? (Let the participant give the time limit)	
Stability indicators		
01	Are AMR surveillance staff available 24/7?	
02	Are there other laboratory staff who enter surveillance data into WHONET in the absence of the facility’s focal person?	
03	Are you trained in detecting and reporting AMR cases?	
04	Have you been trained in the use of WHONET?	
05	Do you have the National AMR Action Plan in physical or electronic copy at the Laboratory?	
06	Do you have the National AMR Control Guide in physical or electronic copy at the laboratory?	
07	Do you have the AMR case notification forms in the laboratory?	
08	Do you have the computer tools necessary to enter the data into the WHONET? (computer, cable, software)	
09	Do you have a stable internet connection for sending data?	
	Are you collecting all priority specimens for surveillance?	Yes or No
	a - Blood	
	b – Stool	
	c - Urethral sample	
	d - Vaginal sampling	
	e - Urine	
10	Have you had any breaks for the notification tools?	
	If YES how long have you been out? a - Less than 1 month. b - Between 1 and 3 months. c - More than 3 months	/________/
Indicators of acceptability		
01	Is the amount of information requested in the notification form acceptable?	
02	Do you hold regular surveillance meetings (at least monthly) in the sentinel surveillance network?	
03	If Yes, are the reports of these meetings available? (check meeting reports)	
04	Are you interested in participating in AMR surveillance?	
05	Is AMR surveillance part of your daily job?	
Data quality indicators (This part is exclusively carried out by the investigator. The latter will have to randomly select 10 sets of data from the available bench/notification sheets. For each set of data, it will be necessary to check that the variables of interest are met correctly.)		
	Number of “filled” variables of interest for all checked records	
01	Age: ______/10	
02	Gender: ______/10	
03	Sampling date: _________/10	
04	Type of sample: _______/10	
05	Department of origin: ________/10	
06	Origin (community or hospital): _________/10	
07	Germ identified: __________/10	
09	Result: _____________/10	
10	Double signature on the bench sheet: _____________/10	
Data transmission completeness indicators		
01	Number of periodic reports transmitted to the higher level during the year 2021	
02	The proportion of periodic reports transmitted during the year 2021 (find out the number of periodic reports expected by the system)	
